# Cell Surface Markers of Mesenchymal Stem Cells: Current Knowledge and Advances in Characterization Technologies

**DOI:** 10.3390/life16010010

**Published:** 2025-12-21

**Authors:** Angelo Santoro, Manuela Grimaldi, Carmen Marino, Enza Napolitano, Michela Buonocore, Anna Maria D’Ursi

**Affiliations:** 1 Department of Pharmacy, University of Salerno, Via Giovanni Paolo II, 132, 84084 Salerno, Italy; asantoro@unisa.it (A.S.);; 2 Department of Chemical Sciences, University of Naples “Federico II”, Complesso Universitario Monte Sant’Angelo, Via Cinthia, 80126 Naples, Italy

**Keywords:** mesenchymal stem cells, surface marker profiling, MSC heterogeneity, stromal subpopulations, phenotypic characterization

## Abstract

Mesenchymal stem cells (MSCs) are pivotal in regenerative medicine due to their high differentiation potential and therapeutic versatility. MSCs are multipotent cells capable of differentiating into adipocytes, chondroblasts, osteoblasts, and, under specific conditions, neural, myocyte, and epidermal lineages. This cell type contributes to tissue repair, immunomodulation, and regenerative therapies for cardiac, orthopedic, and hematological disorders. Accurate identification and characterization of these stem cells are essential for both research and clinical applications. MSCs are typically defined by plastic adherence, expression of surface markers CD105, CD73, and CD90, low or absent expression of hematopoietic markers (CD45, CD34), and in vitro differentiation potential. Understanding the expression patterns and functional relevance of these surface markers is critical for improving isolation strategies, enhancing therapeutic efficacy, and minimizing adverse effects. This review provides a comprehensive overview of the principal surface markers of MSCs, highlighting their significance in stem cell biology and clinical translation.

## 1. Introduction

Regenerative medicine is one of the most recent and innovative branches of medical science, which aims to restore or replace damaged tissues and organs through the combined use of cells, biomaterials, and molecular signaling [1,2]. Current methods may include organ transplantation, which can be autologous or heterologous. Although these types of approaches have been used for a long time, several complications are associated with them. In the case of autologous transplant, for example, problems arise related to the organ harvesting phase, the increase in operating time, and the onset of infections in the transplant site [3]. In order to overcome these problems, medical research is currently focused on developing new technologies and innovative approaches that enable both the identification of more efficient formulations to maximize therapeutic effect and the adaptation for large-scale production [4]. One of the most interesting and modern strategies of regenerative medicine is the use of stem cells, undifferentiated cells present in highly renewable tissues that develop into differentiated cells for the growth or repair of damaged organs [5]. The applications of stem cells in regenerative medicine are broad and expanding. In fact, they can be used to treat a wide range of conditions, including nervous system diseases [6], spinal cord injuries [7], cardiac disorders [8], kidney diseases [9], and immune system disorders [10]. Despite significant progress, there are still issues related to the safety of stem cell-based therapies, like the risk of adverse immune reactions or transplant rejection [11]. Among the various cellular platforms investigated, mesenchymal stem (or stromal) cells (MSCs) have emerged as a cornerstone of regenerative and immunomodulatory therapies due to their multipotent differentiation capacity, high proliferative potential, and paracrine secretory activity. MSCs were initially identified in the bone marrow (BM-MSCs). Still, similar populations have been isolated from a wide array of tissues, including adipose tissue (AT-MSCs), umbilical cord (UC-MSCs), dental pulp (DP-MSCs), placenta and amniotic membrane, synovial membrane, and perinatal tissues [12]. Each tissue source provides MSCs with distinct phenotypic and functional characteristics, reflecting their microenvironmental niche. For instance, BM-MSCs are typically associated with hematopoietic support and osteogenesis, whereas AT-MSCs display enhanced adipogenic and angiogenic potential, and UC-MSCs show robust proliferative capacity coupled with low immunogenicity [13]. Generally, MSCs exhibit a remarkable ability to differentiate into multiple mesodermal lineages, such as osteoblasts, chondrocytes, and adipocytes, and to transdifferentiate into ectodermal or endodermal phenotypes under specific microenvironmental stimuli [14,15]. These results suggest that MSCs are potentially valuable for clinical applications for the treatment of pathologies that currently lack effective therapies [16,17]. An example is their use in cardiac pathologies such as dilated cardiomyopathy and ischemic heart failure, with consequent improvements in cardiac function and capacity, and in quality of life [18,19]. In these treatments, MSCs derived from bone marrow aspiration (autologous or allogeneic) can be administered both locally and intravenously [20]. Another potential therapeutic approach for MSCs is in the orthopedic field, in particular for cartilaginous defects and/or arthrosis, especially of the knee. Currently, the therapy for this type of disease involves a palliative remedy for pain management or a surgical replacement treatment, which, however, is associated with an unfavorable risk/benefit ratio, especially in adult patients. The delivery of bone marrow MSCs, both autologous and allogeneic, can occur through intra-articular injection or replacement of the cartilaginous defect with the use of fibrin polymers. Both approaches report clinical improvement in pain, stiffness, and joint function [21,22,23]. MSCs have also demonstrated a high capacity for immunomodulation and migration towards inflammatory and tumor sites, making them particularly relevant for the treatment of various tumors [24], including urological tumors [25]. Beyond differentiation, MSCs exert potent immunomodulatory effects, achieved through the secretion of cytokines, growth factors, and extracellular vesicles. These factors can modulate T-cell proliferation, promote macrophage polarization, inhibit dendritic cell maturation, and induce regulatory T-cell expansion, thereby contributing to the resolution of inflammation and tissue repair [26,27,28]. Their genetic profile, characterized by low expression of MHC class II molecules and costimulatory proteins, favors their allogeneic and xenogeneic therapeutic use [29]. Given the growing clinical translation of MSC-based products, reproducibility and standardization have become significant challenges. The heterogeneity of MSCs, influenced by factors like tissue source, donor age, and isolation procedures, often leads to variable therapeutic outcomes [30]. Previously mentioned sources include, in addition to MSCs and differentiated lineages, hematopoietic stem cells (HSCs), which are used in regenerative medicine but appear to be less critical because they lack the property of transdifferentiation when placed in other tissues [31]. Therefore, accurate phenotypic characterization is essential to ensure product identity, potency, and compliance with Good Manufacturing Practice (GMP) requirements. Phenotypic profiling through surface marker expression not only validates MSC identity but also enables discrimination from contaminating hematopoietic or endothelial cells [32]. Specific marker combinations may also correlate with functional attributes such as clonogenicity, immunosuppressive potency, and differentiation bias [33]. Consequently, a robust, up-to-date marker panel remains a key prerequisite for translational use. To this aim, in 2006, the International Society for Cellular Therapy (ISCT) proposed minimal criteria for defining human MSCs: (i) plastic adherence under standard culture conditions; (ii) expression of CD105, CD73, and CD90 in ≥ 95% of the population; (iii) lack (≤2%) of hematopoietic markers CD45, CD34, CD14 or CD11b, CD19 or CD79α, and HLA-DR; (iv) trilineage differentiation potential into osteoblasts, adipocytes, and chondrocytes under in vitro conditions [34]. While these criteria provided a foundational framework, they are now considered insufficient to capture the heterogeneity of MSCs. The so-called “canonical markers” (CD105, CD73, CD90) are not MSC-specific, as they are also expressed by fibroblasts, endothelial, and specific epithelial cells [35,36,37]. Moreover, the ISCT panel does not account for functional diversity or in vivo identity, which can vary substantially between tissue sources and physiological states. Recent studies have therefore emphasized the need for new combinatorial markers and functional signatures to identify MSC subpopulations with specific therapeutic profiles. The aim of this review is to provide an overview of canonical and emerging markers, not only to list them by expression profile but also to assign them a functional order in novel strategies for MSC isolation exploiting combinatory panels.

## 2. Phenotypic Identity of MSCs: Overview of Classical Markers

### 2.1. Canonical ISCT Markers: CD105, CD73, and CD90

As established by ICST, the three positive surface antigens CD105, CD73, and CD90 represent the “minimal phenotypic fingerprint” of cultured MSCs. Nevertheless, despite their broad use, each presents significant biological and diagnostic limitations (Table 1).

#### 2.1.1. CD105

CD105, also known as endoglin or egillin, is a type I membrane glycoprotein having a molecular weight of 180 kDa (633 amino acids) that serves as an accessory receptor for TGF-β superfamily ligands. This marker is highly expressed in vascular endothelial cells and in the vasculature of malignant tissues but is less abundantly expressed in monocytes, fibroblasts, chondrocytes, and hematopoietic progenitor cells [44,45]. It is usually found on extracellular matrix proteins and is often involved in cell adhesion [46]. Its structure consists of three distinct regions: an extracellular domain (ECD) of 561 aa, a transmembrane region of 25 aa, and a cytoplasmic tail with variable length [46,47]. The extracellular domain contains N-glycosylation at positions N63, N96, N109, and N282; other glycosylation sites are found between positions 311 and 551, which appear to be abundant in serine and threonine residues [46]. Furthermore, several cysteines are thought to be involved in the dimerization process [45]. Human endoglin presents the arginine-glycine-aspartic acid tripeptide (RGD) absent in mouse endoglin [44,48]. There are two isoforms of CD105, L-CD105 and S-CD105, which differ in the cytoplasmic tail consisting of 47 and 14 amino acids, respectively. Both isoforms are phosphorylated and interact with the growth factor TGF-β. Among these, L-CD105 is the predominant isoform and is commonly expressed on endothelial cells [45]. Numerous studies have demonstrated that CD105 is a marker closely associated with MSCs, although a subpopulation of MSCs is CD105^−^ [49]. Experimental evidence suggests that CD105^+^ MSCs display enhanced capacity to differentiate into osteoblasts and to participate in vascular remodeling, whereas CD105^−^ subsets may exhibit reduced regenerative potency [50,51]. However, recent studies identified CD105^−^ MSCs that also exhibited stronger immune-modulatory capacities than CD105^+^ cells [49,52,53]. Nevertheless, CD105 remains a functional marker reflecting the pro-angiogenic and osteogenic potential of MSCs.

#### 2.1.2. CD73

CD73 (Ecto-5′-nucleotidase, NT5E) is a glycosyl-phosphatidylinositol (GPI)-anchored enzyme that hydrolyzes extracellular AMP into adenosine, a potent immunomodulatory metabolite [54,55]. It is expressed in a wide variety of cell types, including lymphocytes, endothelial cells, smooth muscle cells, epithelial cells, and fibroblasts [56,57,58,59]. In a study conducted by Haynesworth’s research group, it was reported that two monoclonal antibodies (SH3 and SH4), later identified as anti-CD73, showed specificity for MSCs [60]. From this study, it also emerged that SH3 and SH4 did not show reactivity towards hematopoietic cells and osteoblasts, confirming the specificity of CD73 for MSCs. The biological role of CD73 extends beyond metabolism: adenosine generated at the cell surface can suppress T-cell activity, promote M2 macrophage polarization, and regulate vascular tone, mechanisms underlying MSC-mediated immunosuppression and tissue protection [61,62]. Despite its ubiquity, the coordinated expression of CD73 with CD90 and CD105 remains a practical hallmark of cultured MSCs.

#### 2.1.3. CD90 (Thy-1)

Thy-1 or CD90 is a 25–37 kDa, highly glycosylated surface protein. It has a single type V immunoglobulin domain and was originally discovered as a thymocyte antigen [63,64]. The Thy-1 precursor consists of a protein of 161 amino acids that includes three regions: (i) an N-terminal region of 19 residues, called the signal sequence, (ii) a transmembrane domain which includes the region between amino acid residues 20–131, and finally (iii) a C-terminal portion of 31 amino acids (132–161). The active form of CD90 involves an initial cleavage of the transmembrane region, followed by conjugation to the phosphoglyceride GPI. This phospholipid is used by cells during post-translational modifications to anchor proteins to the external portion of the cell membrane, therefore in contact with the extracellular aqueous environment [65]. Although CD90 is frequently included among positive markers for MSCs, its use as an in vivo marker is limited. Indeed, CD90 is a membrane glycoprotein expressed by multiple cell types (e.g., fibroblasts, neurons, certain hematopoietic populations) [66,67,68,69] and therefore lacks specificity for MSCs, thus complicating interpretation of cellular identification in intact tissues [70,71]. Moreover, CD90 expression varies across species and tissues. In several animal models and for certain commercially available antibodies, the epitope is poorly conserved or inefficiently recognized, which limits cross-species comparability and reduces the reliability of in vivo identification [72,73]. Functionally, modulation of CD90 expression has been associated with shifts in differentiation status and immunomodulatory properties: reduced CD90 expression in vitro has been linked to a loss of immunosuppressive activity and an increased propensity toward mesenchymal differentiation, suggesting that CD90 may behave more as a functional regulator than as a strict indicator of “stemness.” These findings indicate that CD90 expression alone is insufficient to identify functional MSC populations in vivo [74,75]. Nevertheless, CD90 may contribute to the identification of specific functional subsets within defined contexts. For example, co-expression of CD90 and CD44 has been associated with increased clonogenicity and adhesion, particularly in dental pulp-derived MSCs [76,77].

### 2.2. CD44: A Bridge Between Phenotype and Function

Although not included in the ISCT criteria, CD44 can be considered as a classical marker. CD44 deserves particular attention thanks to its dual structural and functional relevance. It is a transmembrane glycoprotein with an extracellular hyaluronan-binding domain (HBD) that interacts with hyaluronic acid (HA), a key component of the extracellular matrix [78,79]. CD44-HA binding facilitates MSC adhesion, migration, and homing to injury sites, where HA fragments accumulate during inflammation. Alternative splicing of exons 6–15 generates multiple CD44 variants (CD44v), some associated with tumor progression (e.g., CD44v6) but others implicated in stem-cell niche maintenance [80,81]. In MSCs, CD44 engagement with HA activates signaling cascades that promote cytoskeletal reorganization and secretion of trophic factors. Because of its selective but consistent expression on human MSCs, CD44 represents an attractive molecular target for non-antibody-based isolation strategies [82].

### 2.3. Negative Markers Defining Non-Mesenchymal Phenotypes

To exclude contaminating hematopoietic and immune cells, MSCs must lack surface expression of the following antigens (≤2% of the population): CD34 (hematopoietic progenitors, endothelial progenitors), CD45 (pan-leukocyte marker), CD14/CD11b (monocytic lineage), CD19/CD79α (B-cell lineage) and HLA-DR (MHC class II; inducible under inflammatory stimulation). These “negative markers” ensure minimal hematopoietic contamination but do not distinguish MSC subtypes among different tissue sources. Furthermore, it has been demonstrated that CD34 expression is variable, and therefore MSCs can be either positive or negative for CD34 [83].

## 3. Emerging and Promising Surface Markers for MSCs

Despite the long-standing ISCT criteria, numerous studies have demonstrated that the canonical markers CD105, CD73, and CD90 are insufficient to define functional or tissue-specific MSC subpopulations. Modern single-cell and proteomic analyses have revealed pronounced heterogeneity among MSCs derived from bone marrow, adipose tissue, and perinatal sources [84]. New markers are therefore sought to (i) improve prospective isolation of MSCs in vivo, (ii) identify highly clonogenic fractions, and (iii) distinguish perivascular, neural-like, or immunomodulatory subtypes (Table 2). The following subsections summarize the most relevant markers currently considered promising for research and clinical applications.

### 3.1. CD271 (LNGFR, p75NTR)

CD271, also known as the low-affinity nerve growth factor receptor (LNGFR or p75NTR), is a type I transmembrane receptor belonging to the tumor necrosis factor receptor superfamily. It is strongly expressed on bone marrow-derived MSCs and correlates closely with colony-forming units-fibroblasts efficiency (CFU-F frequency) [95]. CD271^+^ cells represent a rare but highly potent subset capable of trilineage differentiation. In BM aspirates, CD271^+^CD45^−^ cells account for less than 0.1% of total nucleated cells, yet they yield a >100-fold enrichment in CFU-F compared with unselected fractions [96]. Importantly, CD271 is minimally expressed in fibroblasts and hematopoietic progenitors, conferring relative specificity. Its expression declines with in vitro expansion, suggesting that CD271 marks primitive stromal precursors rather than culture-adapted MSCs [97].

### 3.2. CD146 (MCAM)

CD146 (melanoma cell adhesion molecule, MCAM) identifies a perivascular MSC subset located around arterioles and capillaries. CD146^+^ MSCs exhibit high proliferative capacity, enhanced osteogenic differentiation, and elevated expression of angiogenic mediators, including VEGF and ANGPT1 [98]. This marker also correlates with vascular niche localization, as CD146^+^ cells are often pericyte-like and contribute to endothelial stabilization. Functionally, CD146 defines a population with superior multilineage potential and immunosuppressive activity compared with CD146^−^ MSCs. However, CD146 expression can also be detected in endothelial cells, requiring combination with CD271 or PDGFRα for specificity [99,100,101].

### 3.3. Stro-1

Stro-1 was historically among the first MSC-associated antigens, recognized by the monoclonal antibody STRO-1. Stro-1^+^ cells were initially described as CFU-F with mesenchymal potential in bone marrow [86,102]. Although widely used, the precise identity of the Stro-1 antigen remains uncharacterized, and the antibody recognizes a complex carbohydrate or protein epitope on an unknown carrier. Consequently, its use has been declined. Nonetheless, Stro-1 remains a useful pre-enrichment marker when combined with CD146 or CD271 to isolate highly clonogenic MSCs.

### 3.4. GD2 (Disialoganglioside)

GD2 is a sialic acid-containing glycosphingolipid commonly associated with neural tissues and certain malignancies. In 2007, Martinez et al. reported GD2 expression on hBM-MSCs, proposing it as a potential single positive marker for their isolation [87,103]. GD2^+^ MSCs display robust self-renewal and neurotropic properties, showing enhanced differentiation toward neuron-like lineages. Moreover, due to its lipid nature, GD2 is particularly useful for immunofluorescent or flow cytometric sorting and has attracted attention for applications in neural tissue engineering and models of neurodegenerative diseases.

### 3.5. SUSD2 (Sushi Domain-Containing 2)

SUSD2 is a type I transmembrane protein with complement control protein (CCP) domains. Recent studies identified SUSD2 as a reliable marker of clonogenic MSCs in bone marrow and placenta [104,105]. SUSD2^+^ cells exhibit a high frequency of CFU-F, enhanced self-renewal, and multipotency. When combined with canonical markers, SUSD2 allows isolation of MSCs with improved expansion kinetics and reduced senescence [106,107]. Furthermore, SUSD2 has been implicated in cell–matrix interaction and perivascular adhesion, suggesting it marks a subset of stromal progenitors with regenerative potential across multiple tissues.

### 3.6. CD49f (Integrin α6)

CD49f (integrin α6) forms part of the α6β1 integrin complex that mediates adhesion to laminin in the basement membrane. CD49f^+^ MSCs are considered a primitive, stem-like population enriched for self-renewal and proliferative capacity. However, CD49f is not MSC-specific; it is instead expressed on endothelial cells, thymocytes, and platelets. Therefore, CD49f should be interpreted as a stemness-associated rather than lineage-specific marker. When used together with CD271 or CD146, it helps define early stromal progenitors [108,109].

### 3.7. Emerging Markers from Omics-Based Analyses

Recent multi-omics and single-cell transcriptomic studies have revealed a new set of surface candidates associated with MSC identity and functional heterogeneity, including: CD164 (sialomucin involved in cell adhesion and migration; enriched in stromal progenitors); CD200 (immunoregulatory glycoprotein that inhibits macrophage activation; highly expressed in AT- and placental MSCs); CD248 (endosialin) (pericyte marker modulating angiogenesis and matrix remodeling); CD318 (CDCP1) (regulator of cell adhesion and migration; implicated in stress-activated pathways); CD349 (Frizzled-9) (Wnt receptor linked to MSC osteogenic commitment). These molecules provide new combinatorial panels for discriminating between subsets of MSCs with specialized immunomodulatory or regenerative functions. Their expression patterns, uncovered by proteomic and single-cell RNA-seq profiling, support the concept that MSCs constitute a spectrum of lineage-primed stromal progenitors, rather than a single uniform population [110,111,112,113].

### 3.8. Toward Combinatorial Marker Panels

Current evidence suggests that no single marker is sufficient to define MSCs across tissues. Instead, the combination of markers like CD271^+^, CD146^+^, SUSD2^+^, and CD49f^+^ provides a more reproducible definition of CFU-F and therapeutically competent MSCs [88]. Emerging technologies such as CyTOF (mass cytometry) and single-cell RNA-seq are enabling the discovery of multidimensional “MSC signatures”, paving the way for standardized, functionally relevant classification [114].

## 4. Tissue-Specific MSC Markers

Although MSCs from different tissues share a typical core phenotype (CD105^+^, CD73^+^, CD90^+^, CD44^+^, CD29^+^), their functional behavior and surface marker repertoire vary substantially according to anatomical source and microenvironmental cues. The local environment dictates the expression of adhesion molecules, growth factor receptors, and immunomodulatory proteins. Consequently, MSCs derived from bone marrow, adipose tissue, umbilical cord, dental pulp, placenta, and synovial membrane represent distinct subtypes of mesenchymal cells rather than identical cell types. The identification of tissue-specific markers enables refined isolation strategies and provides insight into the biological specialization of each MSC population (Table 3) [115].

### 4.1. Bone Marrow-Derived MSCs (BM-MSCs)

BM-MSCs are the most extensively characterized and remain the reference standard for mesenchymal biology. They typically co-express CD271, CD146, and CD106 (VCAM-1), consistent with a perivascular stromal identity. CD271^+^CD146^+^ subsets represent highly clonogenic CFU-Fs and show robust osteo- and chondrogenic potential. CD106, an adhesion molecule interacting with integrin α4β1 on leukocytes, is upregulated in inflammatory conditions and contributes to hematopoietic stem cell (HSC) niche maintenance. Functionally, BM-MSCs display strong hematopoietic support, osteogenic differentiation, and immunoregulatory capacity, making them optimal for musculoskeletal and immune-related applications [116]. BM-MSCs have also been deeply characterized in terms of their secretome, which includes TGF-β, IL-10, prostaglandin E2, and other mediators that contribute to T-cell suppression and macrophage polarization [117]. Their tri-lineage differentiation potential is among the most consistently validated across species, and BM-MSCs remain the benchmark for evaluating the performance of MSCs derived from other tissues [118]. Nevertheless, their clinical use may be limited by donor age, invasive harvest procedures, and reduced proliferative capacity compared to perinatal MSC sources [119].

### 4.2. Adipose Tissue-Derived MSCs (AT-MSCs)

Adipose tissue provides an abundant and easily accessible source of MSCs with high proliferative potential. AT-MSCs are enriched in CD36, CD10 (neutral endopeptidase), and CD200, reflecting their lipid metabolism and immunomodulatory functions. CD36 mediates fatty acid uptake, linking these cells to adipogenic commitment, while CD10 degrades bioactive peptides such as bradykinin and endothelin. CD200 acts as an immune checkpoint molecule, inhibiting macrophage activation and contributing to the low immunogenicity of AT-MSCs. These cells demonstrate efficient adipogenesis and angiogenesis, suggesting promising roles in soft tissue regeneration, wound healing, and metabolic disease therapy [35,120]. AT-MSCs typically yield a higher number of stem/progenitor cells per gram of tissue compared to BM-MSCs, making them particularly attractive for autologous therapies [121]. In addition to strong adipogenic capacity, they display robust paracrine activity that supports neovascularization and reduces local inflammation [122]. Their immunoregulatory effects include downregulation of TNF-α and IL-6 and the secretion of IL-1Ra, which contributes to their use in inflammatory and ischemic disorders [123].

### 4.3. Umbilical Cord-Derived MSCs (UC-MSCs)

Umbilical cord MSCs, obtained from Wharton’s jelly or perivascular regions, represent a perinatal, developmentally young population with enhanced proliferative and immunosuppressive potential. UC-MSCs typically express CD29 (integrin β1), CD44, CD90, CD105, and CD54 (ICAM-1). The latter mediates leukocyte adhesion and contributes to immunomodulation via cell–cell contact. UC-MSCs exhibit lower MHC expression than adult MSCs, thereby conferring minimal immunogenicity. Their rapid proliferation and scalability make UC-MSCs a preferred source for clinical-grade allogeneic therapies [124,125]. Compared to adult MSCs, UC-MSCs display a more primitive transcriptional profile, enhanced telomerase activity, and superior expansion capacity under GMP conditions [126]. UC-MSCs demonstrate potent suppression of NK cells and effector T cells, favoring their use in immunomodulatory and anti-inflammatory applications, including graft-versus-host disease and autoimmune disorders [127,128].

### 4.4. Dental Pulp-Derived MSCs (DP-MSCs)

Dental pulp MSCs originate from the neural crest and thus exhibit features of both mesenchymal and neuroectodermal lineages. They express CD146, CD24, Stro-1, and CD44, often co-localized with nestin and βIII-tubulin, markers of neurogenic potential. DP-MSCs are particularly responsive to neuronal-inducing media and exhibit enhanced secretion of neurotrophic factors, such as BDNF and NGF. Their combination of regenerative and neuroprotective properties makes them suitable for neural tissue engineering, spinal cord repair, and craniofacial regeneration [129,130]. DP-MSCs also demonstrate high clonogenicity and proliferative rates compared to BM-MSCs and show unique odontogenic differentiation potential, which underlies their role in dentin–pulp complex regeneration [131,132]. Their secretome is enriched in neurotrophic and angiogenic factors, supporting neuronal survival, axonal growth, and vascular remodeling. These characteristics highlight DP-MSCs as a promising cell source for the treatment of peripheral nerve injuries, neurodegenerative conditions, and maxillofacial defects [133,134].

### 4.5. Placental and Amniotic MSCs

The placenta and amniotic membrane represent immunoprivileged, ethically accessible sources of MSCs. Placental MSCs express CD9, CD166 (ALCAM), CD200, and CD340 (HER3), indicating a profile geared toward immune tolerance and epithelial repair. CD9 and CD166 mediate cell–cell adhesion and extracellular vesicle release, while CD200 provides strong immunosuppressive signaling. CD340, a member of the ErbB receptor family, has been implicated in epithelial–mesenchymal interactions and fetal development. Placental MSCs are characterized by broad differentiation potential and robust anti-inflammatory activity, supporting their application in autoimmune disorders and fetal tissue regeneration [135]. Placenta-derived MSCs exhibit high proliferative capacity, low rates of senescence, and strong paracrine activity, producing cytokines and growth factors that support tissue protection and repair [136,137]. Amniotic MSCs share many properties with placental MSCs but additionally display enhanced hepatogenic and neurogenic differentiation potential [136]. Their inherent immune tolerance and reduced expression of co-stimulatory molecules make them highly suitable for allogeneic transplantation and perinatal regenerative therapies [138,139].

### 4.6. Synovial Membrane-Derived MSCs (SM-MSCs)

Synovial MSCs, residing in the subintimal connective tissue of the joint capsule, are characterized by the expression of CD73, CD90, CD105, CD44, and CD106. They display unique mechanotransduction properties, responding to shear stress and mechanical loading through integrin and CD44 signaling. Functionally, SM-MSCs have a strong chondrogenic bias, making them prime candidates for cartilage and osteoarthritis repair. Their secretion of lubricin and extracellular matrix components further supports joint homeostasis [140,141]. SM-MSCs localization within the synovial niche makes them naturally adapted to joint biomechanics and inflammatory environments [141,142]. SM-MSCs have shown promising results in preclinical joint-repair models, including enhanced cartilage regeneration, reduced synovial inflammation, and protection against cartilage degradation in osteoarthritis [143,144,145].

## 5. Markers Associated with Clonogenicity (CFU-F)

### 5.1. Definition and Biological Importance of CFU-F

The CFU-F assay remains one of the most robust functional assays for quantifying the clonogenic potential of MSCs. Initially described by Friedenstein et al. in the 1970s, CFU-Fs represent single progenitor cells capable of extensive proliferation and multilineage differentiation into osteoblasts, chondrocytes, and adipocytes [146,147]. The frequency of CFU-Fs in native tissues is extremely low (typically 1 in 10^4^–10^5^ nucleated cells in bone marrow aspirates). Identifying surface markers that correlate with CFU-F potential has therefore become a key strategy for prospective MSC isolation and potency assessment. Functionally, high CFU-F frequency correlates with enhanced self-renewal, multipotency, and immunomodulatory activity, features essential for reproducible therapeutic efficacy in regenerative medicine (Table 4).

### 5.2. Predictive Markers of Clonogenic MSC Subsets

#### 5.2.1. CD146

CD146 identifies perivascular MSCs with high proliferative and colony-forming capacity. Several studies demonstrated that CD146^+^CD45^−^CD34^−^ stromal cells yield significantly more CFU-Fs than CD146^−^ counterparts [33,88]. CD146^+^ MSCs show elevated expression of vascular genes (ANGPT1, PDGFRB) and stemness-associated markers (OCT4, NANOG), supporting the concept that CD146 expression marks primitive pericyte-like progenitors. Moreover, CD146^+^ subsets retain their differentiation potential even after extended culture, suggesting greater replicative capacity [98,148,149].

#### 5.2.2. CD271

Among all proposed CFU-F markers, CD271 remains the most consistent and specific for bone marrow MSCs. CD271^+^CD45^−^ cells form colonies at high frequency and maintain trilineage differentiation over multiple passages. In combination with CD146, CD271 defines a rare stromal fraction (<0.1%) enriched for skeletal stem/progenitor activity. Functionally, CD271 engagement modulates NF-κB and JNK signaling, influencing survival and proliferation [95].

#### 5.2.3. SUSD2

SUSD2 marks stromal progenitors with exceptional CFU-F efficiency in both bone marrow and placenta. SUSD2^+^CD45^−^ cells show rapid adherence, high colony density, and maintenance of a fibroblast-like morphology during expansion. Notably, SUSD2 expression correlates with clonogenic persistence, defined as the ability of a single cell to give rise to secondary and tertiary CFU-Fs upon replating. This suggests that SUSD2 may serve as a prospective marker of self-renewal within the mesenchymal hierarchy [104,150].

#### 5.2.4. PDGFRα

Platelet-derived growth factor receptor alpha (PDGFRα, CD140α) is a classical mesenchymal receptor tyrosine kinase mediating proliferation and migration. PDGFRα^+^CD51^+^ cells in bone marrow and adipose tissue display high CFU-F frequency and overlap extensively with CD271^+^ populations. PDGFRα signaling promotes MSC proliferation via PI3K/AKT and STAT3 pathways, and PDGFRα^+^ subsets exhibit enhanced trophic support for hematopoietic stem cells (HSCs). Therefore, PDGFRα serves both as a surface identifier and functional regulator of clonogenic stromal cells [151,152].

#### 5.2.5. LEPR

LEPR (CD295) marks a quiescent perivascular stromal subset responsible for sustaining HSC niches in the bone marrow. LEPR^+^ MSCs are potent adipogenic progenitors and contribute to osteogenesis during skeletal remodeling. Single-cell transcriptomic studies revealed that LEPR^+^ stromal cells co-express CXCL12 and SCF (stem cell factor), supporting their role as niche-forming CFU-F progenitors. In situ, LEPR^+^ cells localize around sinusoidal vessels, overlapping partially with CD146^+^ pericytes [153,154].

#### 5.2.6. CXCL12

C-X-C motif chemokine ligand 12 (CXCL12), also known as stromal cell-derived factor 1 (SDF-1), is a secreted chemokine rather than a membrane marker, yet it identifies functionally primitive MSCs with niche-supporting activity. CXCL12-abundant reticular (CAR) cells, originally described in the bone marrow, exhibit high CFU-F frequency and potent HSC-supportive capacity. Although CXCL12 cannot be used directly for sorting, its expression strongly correlates with PDGFRα^+^ and LEPR^+^ stromal populations [155,156].

#### 5.2.7. CD51

CD51 (integrin αV) interacts with multiple β subunits to mediate adhesion to vitronectin and fibronectin in the extracellular matrix. CD51^+^ MSCs, particularly in combination with PDGFRα^+^, represent an enriched clonogenic subset in skeletal tissues. CD51 contributes to cell–matrix crosstalk and regulates osteogenesis via activation of TGF-β signaling, linking it to the differentiation bias of CFU-Fs toward bone-forming lineages [157].

**Figure 1:**
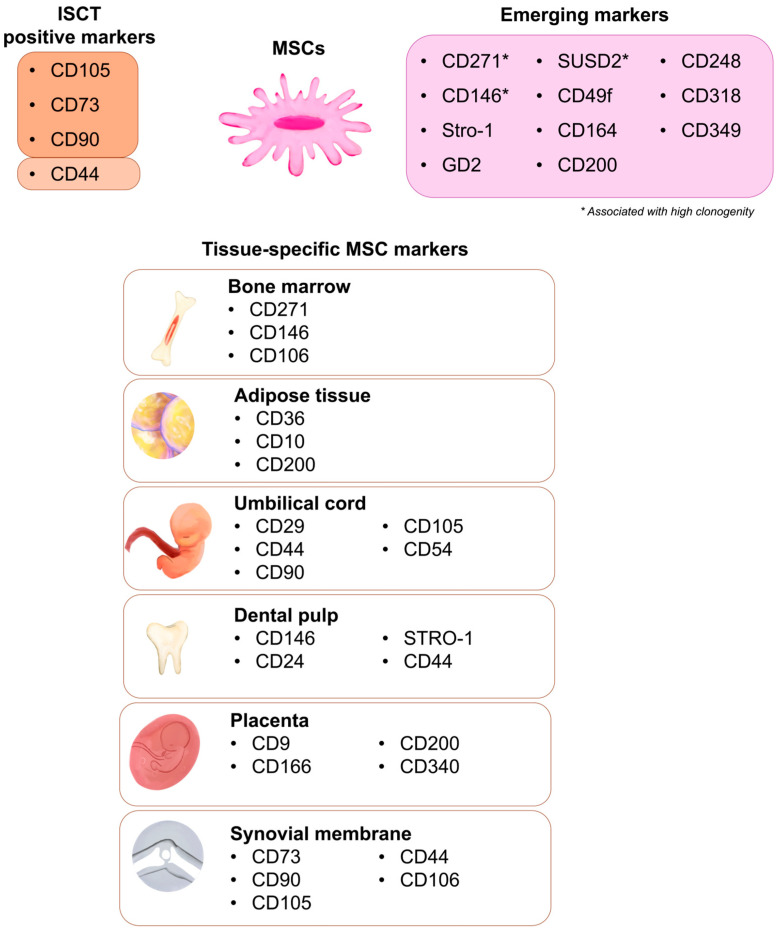
Graphical summary of the most relevant MSC markers.

Understanding CFU-F associated markers is not only of academic value but also critical for standardizing MSC potency assays in translational and GMP contexts. High CFU-F frequency correlates with improved clinical outcomes in bone regeneration, graft integration, and immune modulation. By applying CFU-F enrichment strategies (e.g., CD271^+^CD146^+^ sorting), researchers can generate more homogeneous, high-quality MSC preparations with predictable in vivo behavior. Future efforts will likely combine CFU-F based phenotyping with multi-omic and functional parameters to establish standardized potency metrics for regulatory approval and clinical scalability (Figure 1).

## 6. Novel Methodologies for MSC Identification

Traditional flow cytometric analysis, although indispensable for MSC characterization, provides only a two-dimensional view of a complex and heterogeneous population. The reliance on a few surface antigens, often shared by fibroblasts and endothelial cells, fails to capture the functional and transcriptional diversity of MSCs across tissues and donors. Moreover, culture adaptation profoundly alters marker expression, obscuring the in vivo phenotype [158]. To overcome these limitations, recent advances in single-cell and systems-level omics technologies have enabled multidimensional profiling of MSCs at unprecedented resolution. These tools reveal distinct subpopulations defined by unique transcriptional, proteomic, and functional signatures, supporting a refined, multi-marker–based definition of MSC identity. Single-cell RNA sequencing (scRNA-seq) has revolutionized the understanding of MSC heterogeneity. By capturing the transcriptomes of thousands of individual cells, scRNA-seq allows the reconstruction of lineage hierarchies and the identification of discrete subclusters within heterogeneous MSC populations [159,160,161]. Recent studies have revealed: (i) distinct MSC subsets in bone marrow expressing differential combinations of LEPR, CXCL12, and PDGFRα, corresponding to osteogenic, adipogenic, or niche-forming lineages; (ii) tissue-specific transcriptional programs, where AT-MSCs upregulate lipid metabolism and immune genes, while UC-MSCs display developmental and matrix-related profiles; (iii) senescence-associated subpopulations, identifiable through elevated CDKN2A, IL6, and SASP-related transcripts [162,163,164]. Integrating these datasets has led to the concept of “MSC archetypes”, representing functionally conserved but tissue-adapted stromal progenitors. scRNA-seq data also help refine surface marker panels by linking gene expression (e.g., CD164, CD200, CD248, FRZ9) to distinct functional clusters. While transcriptomic data provide lineage insight, proteomic analyses capture post-transcriptional regulation and surface protein expression, which directly determine cell behavior. Mass spectrometry-based proteomics and label-free quantitative approaches have mapped the MSC proteome across tissues and culture conditions, identifying hundreds of cell-surface and secreted proteins involved in adhesion, signaling, and immunoregulation [165,166]. Notable findings include: (i) upregulation of integrins (αV, α6, β1) and cell adhesion molecules (CD44, CD166) under mechanical stress; (ii) identification of immune-modulatory proteins such as galectin-1, HLA-G, and IDO1, correlating with IFN-γ priming; (iii) proteomic evidence supporting SUSD2, CD200, and CD318 as candidate markers for clonogenic MSC subsets [167,168,169]. Secretomic profiling further refines functional classification, highlighting paracrine mediators (IL-6, TGF-β1, HGF, VEGF) that define immunomodulatory potency. Together, these omics approaches enable functional annotation of MSC subsets beyond surface immunophenotyping. The introduction of mass cytometry (CyTOF) and imaging cytometry has enabled simultaneous quantification of >40 surface and intracellular markers at single-cell resolution [170]. Unlike conventional flow cytometry, which is limited by spectral overlap, CyTOF uses metal isotope–tagged antibodies to generate high-dimensional “cytometric maps.” Applications in MSC research include: multiparametric profiling of donor-to-donor variability in BM- and UC-MSCs; identification of rare subpopulations coexpressing CD271, CD146, and CD49f with distinct signaling signatures; and mapping of activation states during cytokine priming or differentiation using phospho-specific antibodies. Imaging cytometry adds spatial context, visualizing the in situ co-localization of markers such as CD44 and CD105 within tissue niches [171,172,173,174]. These technologies collectively support the establishment of reference cytometric panels for GMP characterization and potency prediction. Combining scRNA-seq, proteomics, and cytometry data through multi-modal integration enables the discovery of robust, cross-platform marker sets that define “MSC subset signatures”. Computational methods, including unsupervised clustering, trajectory inference, and machine learning classifiers, are being applied to predict functional phenotypes from molecular profiles [160,163]. In this context, machine learning–based analyses have successfully distinguished immunomodulatory vs. non-immunomodulatory MSCs based on multi-marker input; predicted differentiation bias (osteogenic, adipogenic, chondrogenic) from transcriptional modules and identified minimal marker panels (e.g., CD271^+^, CD146^+^, SUSD2^+^, PDGFRα^+^) correlating with therapeutic potency [115,175]. This integrative paradigm is reshaping the concept of MSC identity from a fixed set of markers to a data-driven, multi-dimensional signature linked to biological function. Given the variability in marker expression and functional readouts across laboratories, there is an urgent need for standardized, multi-marker panels applicable to clinical-grade MSC products. The current trend is to combine phenotypic markers (CD105, CD73, CD90, CD146, CD271) with functional readouts (CFU-F frequency, secretome analysis, immunosuppressive potency). Regulatory agencies and consortia (ISCT, EBMT, ISO/TC 276) are developing consensus guidelines for advanced MSC characterization, which recommend: multiparametric analysis (≥10 markers) using validated antibodies or label-free alternatives; functional validation for each batch, including CFU-F assay and cytokine profiling; documentation of donor- and tissue-specific expression variability to support batch comparability [176,177]. Standardization of marker panels, potentially informed by machine learning and omic databases, will enhance reproducibility and accelerate regulatory approval of MSC-based therapeutics.

## 7. Practical Applications of MSC Markers

### 7.1. Marker-Guided Selection and Purification of MSCs

Surface markers are not only diagnostic tools but also functional determinants guiding the selection, purification, and standardization of MSC populations for research and clinical use. Two main approaches are commonly employed: Fluorescence-Activated Cell Sorting (FACS) and Magnetic-Activated Cell Sorting (MACS). The first allows high-resolution isolation of subpopulations based on combinatorial marker expression. Although precise, FACS is often limited by low yield, cell stress, and regulatory constraints for clinical manufacturing. The latter offers rapid and scalable selection using magnetic microbeads conjugated to antibodies. MACS is more compatible with GMP protocols but provides lower purity than FACS. By applying these marker-based approaches, it is possible to enrich for CFU-F–rich subsets and to eliminate contaminating hematopoietic or endothelial cells, resulting in more homogeneous and potent MSC preparations [178,179,180]. For example, CD271^+^ selection from bone marrow yields MSC populations with predictable osteogenic differentiation and consistent immunosuppressive function [181]. Similarly, SUSD2^+^ sorting identifies clonogenic stromal cells with superior expansion kinetics and reduced senescence [104].

### 7.2. Impact of Surface Phenotype on Immunomodulatory Properties

The immunoregulatory capacity of MSCs depends strongly on their surface phenotype. Some markers are directly involved in cell–cell interactions and immune checkpoint modulation: CD200, expressed by AT- and placental MSCs, delivers inhibitory signals to macrophages and dendritic cells, reducing pro-inflammatory cytokine production [113]. CD106 (VCAM-1) and CD54 (ICAM-1) mediate MSC binding to activated T cells and promote local immunosuppression via contact-dependent mechanisms [148,182]. PD-L1 (CD274), although not a canonical MSC marker, is inducible under IFN-γ stimulation and correlates with enhanced regulatory function [183]. MSC subsets with high CD146 or CD271 expression exhibit elevated secretion of indoleamine 2,3-dioxygenase (IDO), TGF-β1, and PGE_2_, which are key mediators of T-cell inhibition and macrophage polarization. Thus, phenotypic profiling can predict immunomodulatory potency, enabling selection of the most suitable MSC source for treating autoimmune or inflammatory diseases.

### 7.3. Influence of Markers on Differentiation Potential

Specific markers also correlate with lineage bias and differentiation outcomes: CD146^+^, CD271^+^, BM-MSCs preferentially differentiate toward osteogenic and chondrogenic lineages; CD200^+^ and CD36^+^ AT-MSCs exhibit adipogenic bias, consistent with their metabolic niche; CD146^+^ and STRO-1^+^ DP-MSCs show enhanced neurogenic potential, reflecting their neural crest origin; CD49f^+^ and SUSD2^+^ subsets maintain a “primitive” state with multipotent capacity and delayed senescence. Such correlations support the development of marker-informed differentiation protocols, in which lineage specification can be guided or predicted from surface phenotypes, thereby improving the reproducibility of tissue-engineering outcomes [184].

### 7.4. Marker-Dependent Effects on Therapeutic Efficacy

In vivo, MSCs exert their therapeutic effects primarily through paracrine and immunomodulatory mechanisms, which are influenced by surface phenotype and tissue origin. Preclinical and clinical studies have reported that: CD146^+^CD271^+^ MSCs demonstrate superior bone repair and angiogenesis in fracture models [185]; CD200^+^ placental MSCs show enhanced efficacy in reducing inflammation in graft-versus-host disease (GvHD) [186]; UC-MSCs, characterized by CD54^+^ and low HLA-DR expression, have produced favorable outcomes in autoimmune and inflammatory indications such as Crohn’s disease and multiple sclerosis [127]. Furthermore, CD44-mediated homing to HA-rich inflamed tissues contributes significantly to MSC localization and function after systemic administration [187,188]. Therefore, rational selection of marker-defined MSC subtypes can optimize therapeutic performance across disease contexts [189].

### 7.5. Pittenger Protocol

**Figure 2:**
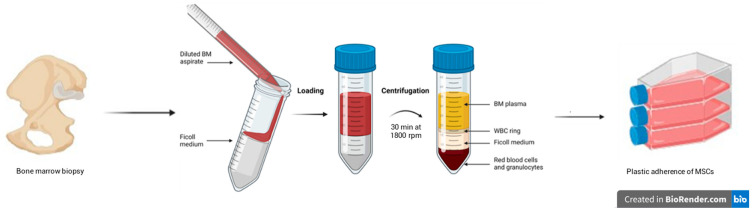
General scheme of the Pittenger protocol (figure created in BioRender.com).

The isolation methods described above can be used individually or in combination, as occurs in the Pittenger protocol (Figure 2) [190]. Specifically, this protocol involves an initial centrifugation phase using a density gradient: the Ficoll polymer is dissolved in a saline solution in a centrifuge tube, to which a sample of cells from the aspirate is added. Following centrifugation, cells separate based on their density: lighter, less dense cells are usually collected at the top of the tube, while heavier, denser cells are collected at the bottom.

The phase following gradient separation is selective adhesion, which will allow the removal of non-adherent cells (including the majority of HSCs); finally, in the last phase, antibodies will be used to improve the homogeneity of the population through the phenotypic characterization of the MSCs through the recognition of the positive markers indicated by the ISCT [72,88]. The main disadvantage of this protocol is the use of antibodies for the selection of MSCs.

## 8. Limitations in the Use of Antibodies

Since the first recombinant immunoglobulin (Ig) was developed in 1984, modern science has relied on antibodies for the detection of biomolecules, given their affinity for specific epitopes [191,192]; this allows them to be ideal reagents in a wide range of biological assays: immunoassays such as ELISA, Western blot, flow cytometry, immunoprecipitation, and many others. Antibodies are glycoproteins characterized by a symmetric structure composed of two light chains (VL) with a molecular weight of approximately 50 kDa, and two heavy chains (VH) with a molecular weight of approximately 25 kDa, covalently linked by disulfide bridges; each chain is made up of an amino-terminal variable region (Fab) and a carboxy-terminal constant region (FC), also known as a crystallizable fragment. The variable regions of both chains form the antigen-binding site, and since each immunoglobulin is composed of two VL and two VH chains, there will be two binding sites. The FC regions, however, do not interact directly with the antigen but instead interact with molecules and effector cells of the immune system, and they are responsible for a large part of the biological functions carried out by antibodies. Immunoglobulins are mainly produced by hybridoma technology. This process involves various steps: the first phase is immunization, i.e., the injection into laboratory animals, mostly mice or rabbits, of the antigen against which antibodies are to be produced; after several injections there will be an overproduction of B cells in the animal, which will then be removed from the spleen and fused with myeloma cells, thus obtaining the hybridoma which possesses both the ability to produce antibodies typical of B lymphocytes, as well as longevity and unlimited reproductivity typical of myeloma. The hybridomas obtained are placed in multi-well plates so that each well contains only one cell, and antibody production occurs in each well. Subsequently, we proceed to a screening phase to select hybridomas that produce the desired antibodies, which will then be transferred to cell culture flasks [193,194]. Although the effectiveness of immunoglobulins has been widely approved, several drawbacks can be encountered in their use in therapy: (i) long production times and high costs: the production process of hybridomas is rather laborious and with low yields, as well as being expensive in economic terms due to the high cost of the raw materials used; (ii) selectivity of many commercially available antibodies often questionable: Pharmaceutical companies typically assign catalog numbers to antibodies based on the immunizing antigen and how the antibody was produced; however, several antibodies against a target protein are often available with little information provided regarding their affinity or specificity [195]; (iii) unfavorable pharmacokinetics: antibodies have reduced tissue penetration due to their high molecular weight [196]; (iv) risk of immunogenicity: the immunoglobulins used are mostly murine, chimeric or humanized IgG, so they can overstimulate the immune system and trigger strong immune responses, such as cytokine release syndrome [197]. In order to overcome these limitations, while maintaining the advantage of using monoclonal antibodies, i.e., the high selectivity towards surface proteins, non-Ig ligands have been developed, i.e., engineered molecules of various types which present multiple advantages and applications.

### 8.1. Affibodies

Affibodies are small proteins (about 6.5 kDa) deriving from the B domain of staphylococcal protein A [198]. The B domain is a relatively short portion, composed of about 58 amino acids, typically devoid of residues of cysteine and therefore stable in the redox environment of the cell [199]. This portion is folded into a three-helix bundle structure and exhibits one of the fastest folding kinetics ever reported [200]. The folding properties, together with the thermal stability and high solubility, have contributed to increasing scientific interest in this domain. The B domain was initially mutated at key positions to improve chemical stability, and the resulting engineered variant was named the Z domain [201]. The Z domain maintains affinity for the Fc portion relative to the antibody, whereas the weaker affinity for the Fab region is almost wholly lost. Based on this strategy, affibody molecules have been generated that show specific binding to a wide variety of proteins; this characteristic makes these molecules useful for imaging, diagnostic, and therapeutic applications [199,202]. An example of an application of these molecules is in tumor diagnostics as imaging tracers; in fact, affibodies have been synthesized that recognize and bind specific tumor proteins, such as HER2. In this procedure, an engineered affibody (ABY-025) with high affinity for human epidermal growth factor receptor 2 (HER2) was administered for the in vivo detection and characterization of HER2- positive in patients with metastatic breast cancer [203,204]. In fact, with the production of affibodies, the aim was to combine the molecular recognition properties, already known in antibodies, with improved characteristics, such as small dimensions that allow their production by a standard peptide synthesis protocol and high stability.

### 8.2. Single-Chain Variable Fragments

Single-chain fragments (scFv, 25 kDa) are fusion proteins composed of the variable regions of the heavy (VH) and light (VL) antibody chains, connected by a linker peptide approximately 15 amino acids long that allows interaction between the two chains. scFvs are successfully isolated mainly from bacterial cell cultures such as Escherichia coli [205,206], but also from mammalian or yeast cells [207], plants [208], and insects [209]; the production of these molecules using the systems mentioned constitutes an enormous advantage compared to the production of monoclonal antibodies by hybridoma cells. Furthermore, their relatively small size compared to IgG also suggests greater ease of penetration into tissues and greater clearance from the blood, which is why they find wide application in therapeutic and diagnostic fields [205]. scFv fragments can be fused to a variety of toxins such as cytotoxic proteins [210], radionuclides [211] or drugs [212]. The immunotoxins produced could deliver their agents specifically to cells that present the tumor antigen. An example of an immunotoxin currently generated for the treatment of fetal neoplasms is given by the fusion of a fully human Fab fragment with Pseudomonas aeruginosa exotoxin A to generate an anti-fAChR immunotoxin (scFv35-ETA) [213]. scFvs also include diabodies, scFv dimers characterized, therefore, by two binding sites for the antigen.

### 8.3. Nanobodies

Nanobodies, also known as single-domain antibodies (sdAb), are fragments derived from heavy-chain IgG antibodies found in the Camelidae family [214]. Nanobodies have a molecular weight between 12 and 15 kDa and are therefore significantly smaller than an antibody (150–160 kDa) and even the antigen-binding region (Fab, approximately 50 kDa) and scFv (approximately 25 kDa). Due to their size, simple structure, high antigen-binding affinity, and remarkable stability under extreme conditions, nanobodies have the potential to overcome many of the limitations of monoclonal antibodies [215]. Also, in this case, the advantages of producing these molecules enable imaging and therapeutic applications across a wide range of pathologies. A relatively new application in which nanobodies have been successfully used is biosensor development (devices with a specific bio-receptor for a target antigen fixed to a semiconductor), since their ability to withstand high temperatures and pH changes allows storage in non-ideal environments [216]. An example of a biosensor using a nanobody is the one used for the detection of fibrinogen, a biomarker for cardiovascular disease [217]. It has also been shown that they are excellent candidates for the development of biosensors to detect SARS-CoV-2 Spike proteins [218,219,220].

### 8.4. Monobodies

Monobodies are synthetic proteins (10 kDa) designed using the 10th fibronectin type III (FN3) domain of human fibronectin conjugated to a variable region as a scaffold. This association was originally seen to occur via an “RGD” (Arg-Gly-Asp) amino acid portion of FN3 [221]. Unlike conventional antibodies, monobodies are engineered to mimic antigen binding without relying on the immunoglobulin variable domain fold, instead using exposed loops on a simpler β-sandwich scaffold that provides structural robustness with fewer folding constraints [222]. Although they were originally designed to functionally resemble nanobodies, they offer unique structural advantages, such as a smaller size (20–25% smaller) and a compact protein core lacking disulfide bonds [223,224]. The absence of disulfide bonds improves stability in reducing environments, such as the cytosol, and facilitates robust protein production in both prokaryotic and eukaryotic hosts [225]. This simplified architecture also allows diversification of surface loops without destabilizing the core scaffold. Their small size (<100 amino acids), simple structure, and relative stability allow them to be expressed in many cell types, both eukaryotic and prokaryotic. Furthermore, the fibronectin type III scaffold presents multiple “association zones” across β-sheet surfaces and loops that can be engineered to bind targets with high affinity, sometimes with binding surfaces not readily accessible to conventional antibody fragments [226]. Due to their ability to be engineered to bind a variety of proteins of interest, they have become attractive tools for biomedical research and biotechnology [227,228]; furthermore, they also prove very promising for the treatment of autoimmune diseases [229], for tumors [225], and, more recently, for SARS-CoV-2 [230,231]. Monobodies can achieve nanomolar binding affinities comparable to those of antibodies but with advantages in tissue penetration and reduced immunogenicity owing to their small size and simplified fold [232]. Their ability to be engineered into multivalent or fusion constructs further expands their functional versatility in both research and therapeutic settings [221,233].

### 8.5. Peptide Aptamers

**Figure 3:**
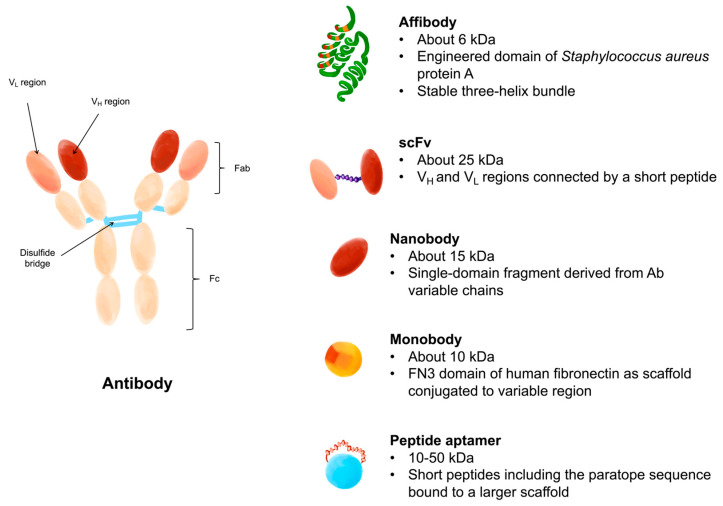
Scheme of antibody compared to non-Ig ligands.

Peptide aptamers (PA) are short peptide sequences (5–20 amino acids) functionalized with different protein scaffolds and can selectively bind to a target protein, inactivating it [234,235]. PAs are small molecules (10–50 kDa) characterized by high stability and solubility, and are produced by chemical synthesis or bacterial expression; therefore, they are available in large quantities [236]. The applications of these antibody mimetics are potentially unlimited, especially in fields where immunotherapy is gaining significant attention, as they are safe and antigen-specific [237]. However, the potential immunogenicity induced by engineered scaffolds derived from non-human proteins and the low tissue penetration, due to their size, are potential drawbacks that must be taken into consideration [238]. Although several studies have demonstrated that the binding affinity of functionalized aptamers can be up to 1000 times higher than that of free peptides [207], short free peptide sequences are sometimes preferred as alternatives to monoclonal antibodies. The latter were considered non-pharmacological molecules for a long time but are now being reconsidered, as they are chemically stable, biocompatible, easy to synthesize, and have reduced production costs [239,240,241]. A rather recent application of peptide ligands is their use in the treatment of Alzheimer’s disease; they have been widely studied as potential anti-amyloid molecules [242,243,244,245,246] or as molecules capable of interfering with the formation of amyloid plaques, the etiological agent of the pathology [247]. The design of short highly specific peptide sequences towards a target exposed on a cell surface is made possible by the analysis of the macromolecular complex, which allows such sequences to be derived by evaluating the antibody/antigen interactions that are established at the moment where the complex is formed [248,249,250,251]. The choice of residues that characterize the peptide sequences, so that they can simulate the paratopes, is supported by knowledge of a certain number of 3D coordinates deposited in the Protein Data Bank (http://www.wwpdb.org/ accessed on 12 May 2025) for Fab or other antibody analogues in complex with their antigen. However, many obstacles arise in the development of these peptide sequences, the most important are linked to the availability of crystallographic structures of a limited number of immunoglobulins deposited in databases and, to the difficulty of synthesizing and testing the efficacy of a large number of sequences in a short period of time (Figure 3).

## 9. Discussion

The growing complexity revealed in mesenchymal stem cell biology makes it increasingly clear that the traditional reliance on a narrow set of canonical markers is no longer adequate to define the full spectrum of bone marrow-derived MSC identity. At the same time, it is important to acknowledge that the classical criteria established by the ISCT remain fundamental and operationally crucial for the initial identification and standardization of MSC populations, especially in regulatory and manufacturing settings. Rather than representing a uniform stromal entity, MSCs emerge as a group of lineage-primed, functionally heterogeneous progenitors shaped by their developmental niche, environmental cues, and dynamic interaction with the immune and vascular systems. In this framework, classical functional assays define a minimal common denominator of “MSC-like” behavior, whereas emerging phenotypic and molecular approaches seek to resolve biologically meaningful diversity within this baseline population. This conceptual shift has profound implications for how MSCs are isolated, characterized, and ultimately deployed in clinical settings. As evidence accumulates, surface markers such as CD271, CD146, SUSD2, PDGFRα, and LEPR not only refine phenotypic profiling but also illuminate distinct biological trajectories, ranging from perivascular progenitors with high clonogenicity to niche-supporting stromal subsets with strong immunomodulatory capacity. Importantly, these markers do not invalidate classical MSC definitions but rather provide an additional layer of resolution that links surface phenotype to in vivo localization, lineage bias, and functional specialization. These insights challenge the adequacy of the minimal ISCT criteria and underscore the need for multidimensional marker panels that better capture in vivo identity and functional potential. Equally transformative has been the integration of omics-based technologies, which reveal MSC heterogeneity at an unprecedented resolution. Single-cell transcriptomics, proteomics, and high-parameter cytometry collectively demonstrate that surface phenotype is tightly woven into the molecular networks governing immunoregulation, differentiation bias, senescence, and trophic activity. As a result, phenotypic signatures are no longer viewed as static descriptors but as dynamic predictors of therapeutic behavior. This emerging framework provides a scientific rationale for marker-guided enrichment strategies that favor highly clonogenic, potent subsets. Such strategies build upon, rather than replace, established functional assays, thereby improving manufacturing consistency and reducing functional variability, two major barriers in translating MSC-based therapies to predictable clinical outcomes. The increasing availability of multi-modal datasets also paves the way toward standardized potency assays anchored in biology rather than convenience. By correlating surface markers with CFU-F frequency, secretome composition, and immunoregulatory function, it becomes feasible to develop robust identity-potency relationships that can be integrated into GMP workflows. Such an approach will be essential for regulatory harmonization and for ensuring that cell therapy products derived from different donors, tissues, or manufacturing platforms remain comparable in quality and efficacy. Finally, the evolution of MSC marker research is reshaping not only how these cells are defined, but also how they are conceptualized as therapeutic agents. The field is moving toward a model in which MSCs are stratified into rationally selected subpopulations with tailored functional attributes, rather than used as broadly defined stromal isolates. This transition does not abandon classical MSC concepts but reframes them as an essential entry point into a more precise and biologically informed taxonomy. This progression toward precision stromal therapeutics guided by surface phenotype, functional genomics, and microenvironmental context, promises to enhance reproducibility, potency, and safety across clinical applications. Continued integration of high-resolution technologies with mechanistic studies will be essential for establishing a unified and biologically coherent framework for MSC identity, enabling the next generation of targeted and standardized cell-based therapies.

## Figures and Tables

**Table 1:** Overview of canonical MSC surface markers’ biological role, specificity, and limitations.

Marker	Biological Role	Expression Profile	Specificity for MSCs	Notes
**CD105 (Endoglin)**	TGF-β co-receptor; regulates angiogenesis and osteogenesis	Endothelial cells, MSCs, fibroblasts	Moderate	Expressed in endothelium and tumors; up-regulated in inflammation [38]
**CD73 (NT5E)**	Catalyzes AMP in adenosine; immunomodulation	MSCs, lymphocytes, endothelial cells	Low/moderate	Broad expression; up-regulated in cancer and ischemia [39,40].
**CD90 (Thy-1)**	Cell adhesion and signaling; fibroblast activation	MSCs, neurons, fibroblasts	Low	Species variability; not preserved across mammals [41].
**CD44**	HA receptor; migration, homing	MSCs, leukocytes, epithelial cells	Moderate	Multiple splice variants; context-dependent [42].
**CD34/CD45/CD14/CD19/HLA-DR**	Hematopoietic markers	Blood, immune cells	Negative identifiers	May be transiently expressed in early progenitors [43].

**Table 2:** Emerging MSC surface markers and their biological significance.

Marker	Biological Role	Expression Profile	Specificity for MSCs	Notes
**CD271 (LNGFR)**	Regulates neural growth, survival, and apoptosis.	BM-MSC	Moderate	High clonogenicity, hematopoietic support.
**CD146 (MCAM)**	Cell adhesion; promotes migration and angiogenesis	Perivascular MSCs	Low/moderate	Pericyte-like MSCs; high immunomodulation.
**Stro-1**	Undefined role	BM-MSC	High	Considered the most known MSC marker [85]. However the Stro-1 antigen has not been identified yet [86].
**GD2**	Involved in cell adhesion and signaling	BM-MSC, neural-like MSCs	High for BM-MSCs.	Promising as a single marker to isolate MSCs from bone marrow [87]. However, a portion of BM-MSCs CD34^+^ or CD19^+^ cells also express GD2 [88].
**SUSD2**	Involved in cell adhesion and immune modulatory functions	BM, placenta	Moderate	It can promote the enrichment of CFU-F colonies if combined with canonical MSC markers [89].
**CD49f (Integrin α6)**	Laminin receptor	BM-MSC, AT-MSC	Low	Primitive progenitors; limited specificity [88].
**CD164**	Sialomucin regulating adhesion, migration and proliferation	BM-MSC	Moderate	Stromal progenitor enrichment [90].
**CD200**	Immunoregulation	AT-MSC, placental MSC	Low/moderate	Inhibits macrophage activation [91].
**CD248 (Endosialin)**	Tissue remodeling, angiogenesis	Pericytes	Low	Vascular regeneration marker [92].
**CD318 (CDCP1)**	Adhesion and migration	BM-MSC	Moderate	Stress-response and repair [93].
**CD349 (Frizzled-9)**	Wnt receptor mediating wnt/β-catenin signaling	BM-MSC	Moderate	Wnt-dependent osteogenesis [94].

**Table 3:** Tissue-specific MSC markers and biological/functional notes.

Tissue of Origin	Distinctive Markers	Biological/Functional Notes
**Bone Marrow (BM-MSCs)**	Commonly associated with CD271^+^, CD146^+^, CD106^+^	High CFU-F frequency; perivascular niche; osteogenic and hematopoietic support.
**Adipose Tissue (AT-MSCs)**	Typically enriched in CD36^+^, CD10^+^, CD200^+^	Strong adipogenic and angiogenic potential; high immunomodulation.
**Umbilical Cord (UC-MSCs)**	Commonly express CD29^+^, CD44^+^, CD90^+^, CD105^+^, CD54^+^	High proliferation, low immunogenicity; suitable for allogeneic use.
**Dental Pulp (DP-MSCs)**	Often associated with CD146^+^, CD24^+^, Stro-1^+^, CD44^+^	Neurogenic potential; high responsiveness to neural induction.
**Placenta/Amniotic MSCs**	Frequently enriched in CD9^+^, CD166^+^, CD200^+^, CD340^+^	Strong immunosuppressive profile; fetal tissue regeneration.
**Synovial MSCs**	Commonly express CD73^+^, CD90^+^, CD105^+^, CD44^+^, CD106^+^	Mechanotransductive properties; chondrogenic potential.

**Table 4:** Markers associated with high CFU-F potential and their biological significance.

Marker	Main Association	Functional Correlate	Biological Role/Notes
**CD146 (MCAM)**	Perivascular MSCs	High proliferation, angiogenesis	Primitive, multipotent progenitors.
**CD271 (LNGFR)**	BM stromal progenitors	CFU-F enrichment, skeletal stem cells	Highly specific for BM-MSCs.
**SUSD2**	BM, placenta	Self-renewal, secondary colony formation	Marker of clonogenic persistence.
**PDGFRα**	BM, AT	Proliferation, trophic support	Growth factor signaling; HSC niche regulation.
**LEPR (CD295)**	BM perivascular	Niche-forming activity	Supports HSCs; osteo-adipogenic lineage.
**CXCL12 (SDF-1)**	CAR stromal cells	Chemotaxis, HSC retention	Secreted factor; identifies niche MSCs.
**CD51 (Integrin αV)**	Skeletal progenitors	Adhesion, osteogenesis	TGF-β activation; osteogenic bias.

## Data Availability

No new data were created or analyzed in this study. Data sharing does not apply to this article.

## Abbreviations

The following abbreviations are used in this manuscript:

BM-MSCs	bone marrow-derived mesenchymal stem cells
MSCs	mesenchymal stem cells
AT-MSCs	adipose tissue mesenchymal stem cells
UC-MSCs	umbilical cord mesenchymal stem cells
DP-MSCs	dental pulp mesenchymal stem cells
GMP	good manufacturing practice
ISCT	international society for cellular therapy
ECD	extracellular domain
GPI	glycosyl-phosphatidylinositol
HBD	hyaluronan-binding domain
HA	hyaluronic acid
LNGFR	low-affinity nerve growth factor receptor
CFU-F	colony-forming units-fibroblasts
MCAM	melanoma cell adhesion molecule
CCP	complement control protein
HSC	hematopoietic stem cell
SM-MSCs	synovial membrane-derived MSCs
CXCL12	CXC motif chemokine ligand 12
scRNA-seq	single-cell RNA sequencing
FACS	fluorescence-activated cell sorting
MACS	magnetic-activated cell sorting
IDO	indoleamine 2,3-dioxygenase
GvHD	graft-versus-host disease
HER2	human epidermal growth factor receptor 2
scFv	single-chain fragments
sdAb	single-domain antibodies
PA	peptide aptamers
